# Circulating metabolic signatures of rapid and slow progression to type 1 diabetes in islet autoantibody-positive children

**DOI:** 10.3389/fendo.2023.1211015

**Published:** 2023-09-06

**Authors:** Santosh Lamichhane, Partho Sen, Alex M. Dickens, Matilda Kråkström, Jorma Ilonen, Johanna Lempainen, Heikki Hyöty, Riitta Lahesmaa, Riitta Veijola, Jorma Toppari, Tuulia Hyötyläinen, Mikael Knip, Matej Orešič

**Affiliations:** ^1^ Turku Bioscience, University of Turku and Åbo Akademi University, Turku, Finland; ^2^ Department of Chemistry, University of Turku, University, Turku, Finland; ^3^ Immunogenetics Laboratory, Institute of Biomedicine, University of Turku, Turku, Finland; ^4^ Department of Pediatrics and Adolescent Medicine, Turku University Hospital, Turku, Finland; ^5^ Clinical Microbiology, Turku University Hospital, Turku, Finland; ^6^ Faculty of Medicine and Life Sciences, University of Tampere, Tampere, Finland; ^7^ Fimlab Laboratories, Pirkanmaa Hospital District, Tampere, Finland; ^8^ InFLAMES Research Flagship Center, University of Turku, Turku, Finland; ^9^ Institute of Biomedicine, University of Turku, Turku, Finland; ^10^ Department of Pediatrics, PEDEGO Research Unit, Medical Research Centre, University of Oulu, Oulu, Finland; ^11^ Department of Children and Adolescents, Oulu University Hospital, Oulu, Finland; ^12^ Institute of Biomedicine, Centre for Integrative Physiology and Pharmacology, and Centre for Population Health Research, University of Turku, Turku, Finland; ^13^ School of Science and Technology, Örebro University, Örebro, Sweden; ^14^ Research Program for Clinical and Molecular Metabolism, Faculty of Medicine, University of Helsinki, Helsinki, Finland; ^15^ Department of Pediatrics, Tampere University Hospital, Tampere, Finland; ^16^ School of Medical Sciences, Faculty of Medicine and Health, Örebro University, Örebro, Sweden

**Keywords:** birth cohort, lipidomics, metabolomics, type 1 diabetes mellitus, gut microbial metabolites

## Abstract

**Aims/hypothesis:**

Appearance of multiple islet cell autoantibodies in early life is indicative of future progression to overt type 1 diabetes, however, at varying rates. Here, we aimed to study whether distinct metabolic patterns could be identified in rapid progressors (RP, disease manifestation within 18 months after the initial seroconversion to autoantibody positivity) *vs.* slow progressors (SP, disease manifestation at 60 months or later from the appearance of the first autoantibody).

**Methods:**

Longitudinal samples were collected from RP (n=25) and SP (n=41) groups at the ages of 3, 6, 12, 18, 24, or ≥ 36 months. We performed a comprehensive metabolomics study, analyzing both polar metabolites and lipids. The sample series included a total of 239 samples for lipidomics and 213 for polar metabolites.

**Results:**

We observed that metabolites mediated by gut microbiome, such as those involved in tryptophan metabolism, were the main discriminators between RP and SP. The study identified specific circulating molecules and pathways, including amino acid (threonine), sugar derivatives (hexose), and quinic acid that may define rapid *vs*. slow progression to type 1 diabetes. However, the circulating lipidome did not appear to play a major role in differentiating between RP and SP.

**Conclusion/interpretation:**

Our study suggests that a distinct metabolic profile is linked with the type 1 diabetes progression. The identification of specific metabolites and pathways that differentiate RP from SP may have implications for early intervention strategies to delay the development of type 1 diabetes.

## Introduction

Type 1 diabetes is an autoimmune disease that arises due to the destruction of the insulin-producing beta cells in the islets of the pancreas (1). Many exogenous factors have been associated with the development of type 1 diabetes, including viral infections, diet, exposure to toxins and chemicals, and gut microbiota (2–4). The preclinical period of type 1 diabetes is characterized by serum islet autoantibodies. Children who develop multiple (≥2) autoantibodies increase the likelihood of clinical type 1 diabetes later in life (5–7), but at varying rate (8). Age at the time of seroconversion and high titers of insulin autoantibodies (IAA) influence the progression rate (9, 10).

Metabolomics studies focusing on a possible link between type 1 diabetes and the circulating metabolome suggest that metabolic dysregulation precedes the clinical presentation of diabetes, prior to an asymptomatic pre-diabetes period (11–13). However, metabolomes of rapid *vs.* slow progressors to type 1 diabetes following the seroconversion have not yet been investigated. Here we analyzed circulating polar metabolites (‘metabolomics’) and molecular lipids (‘lipidomics’) from serum samples obtained from children recruited into the Finnish Type 1 Diabetes Prediction and Prevention study (DIPP), divided into two study groups: children who progressed slowly to overt disease (SP, duration from initial seroconversion to clinical disease ≥ 60 months and children who progressed rapidly (RP) to type 1 diabetes (time from seroconversion to clinical disease ≤ 18 months).

## Methods

These methods are adapted versions of descriptions in our related work (14).

### Study design and protocol

**Figure 1:**
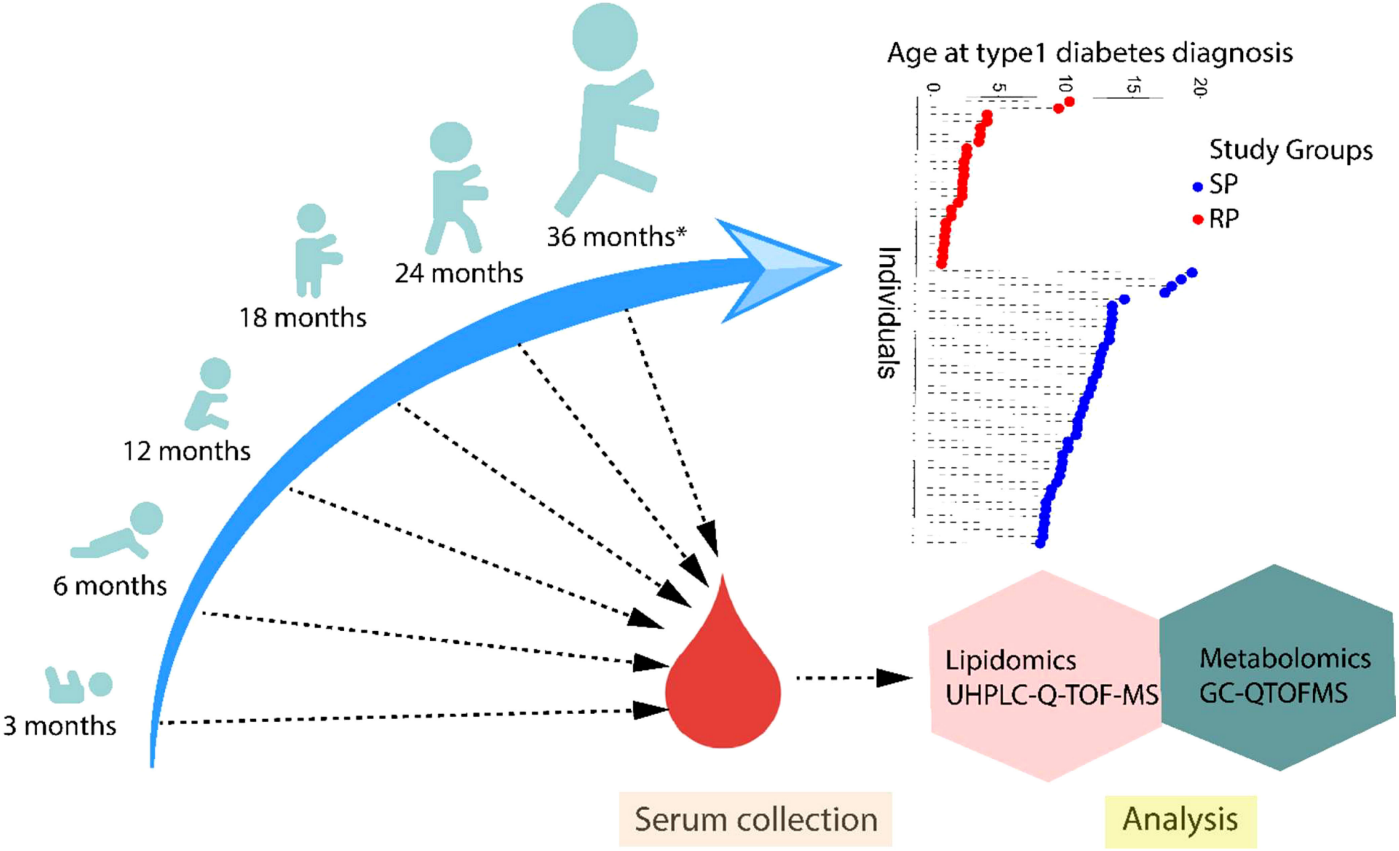
An overview of the study design. The study cohort comprised children rapidly progressing to overt type 1 diabetes (RP) and children slowly progressing to clinical disease (SP) during the follow-up until the age of 15 years. For each child, longitudinal serum samples were drawn, corresponding to the age of 3, 6, 12, 18, 24 or ≥36 months. Moreover, the age at diagnosis of type 1 diabetes is shown in the participating children.

In this study, the samples were obtained from the Finnish Type 1 Diabetes Prevention and Prediction Study (DIPP) (15), an observational birth cohort study following the participants from the age of 3 months to 15 years of age or to the diagnosis of type 1 diabetes. The DIPP study was initiated in 1994 and is currently running in three university hospitals in Finland (Oulu, Tampere, and Turku). The participants involved in the current study were chosen from the subset of DIPP children who were born in Turku University Hospital since sequential unthawed serum samples, collected between 1998 and 2012 and stored at -80°C were available from these participants. The study protocol was approved by the ethics committee of the Hospital District of Southwest Finland. The study was conducted according to the guidelines in the Declaration of Helsinki. All families provided written, informed consent for participation in the study. For each child, longitudinal samples for metabolomics analysis were obtained at the age of 3, 6, 12, 18, 24 or ≥ 36 months (**Figure 1**).

This study included samples (n=239 for lipidomics and n=213 for polar metabolites) from 66 children, divided into two groups, being 25 RPs and 41 SPs. Selected characteristics of the participants in this study are listed in **Table 1**. In this study, non-fasting blood samples were collected, serum was prepared within 3 hours of sample collection and stored at -80°C until analyzed.

**Table 1:** Demographic characteristics of study population.

	RP (n= 25)	SP (n=41)
Gender (girls, boys)	(12, 13)	(19, 22)
Age at time of diagnosis (mean ± SD)	2.88 ± 2.34	11.84 ± 2.80
Age at time of first seroconversion (mean ± SD)	2.81 ± 2.28	1.99 ± 1.19
Interval from seroconversion to type1 diabetes (mean ± SD)	0.70 ± 0.37	9.86 ± 2.53

### HLA genotyping

Screening for HLA-conferred susceptibility to type 1 diabetes was carried out using cord blood samples. The HLA-genotyping was performed with a time-resolved, fluorometry-based assay for four alleles using lanthanide chelate-labelled, sequence-specific oligonucleotide probes detecting DQB1*02, DQB1*03:01, DQB1*03:02, and DQB1*06:02/3 alleles (16). The carriers of DQB1*02/DQB1*03:02 or DQB1*03:02/x genotypes (here x≠ DQB1*03:01, DQB1*06:02, or DQB1*06:03 alleles) were categorized as children with increased HLA susceptibility to type 1 diabetes and accordingly invited to the DIPP follow-up program.

The study participants underwent more extensive HLA genotyping. This genotyping defined all common European HLA-DR-DQ haplotypes at low resolution and at higher resolution haplotypes where this was relevant for the assessment of the risk for type 1 diabetes, e.g. HLA-DR4 subtypes in DR4-DQ8 haplotypes. In a series of 2,991 family trios from the Finnish Pediatric Diabetes Register, the genotype risks were defined and genotypes were combined into six groups from O (strongly protective) to 5 (high risk) which did not overlap for 95% confidence intervals of their OR values for type 1 diabetes (17).

### Detection of islet autoantibodies

The children with HLA-conferred genetic susceptibility were prospectively observed for levels of diabetes-associated autoantibodies [islet cell antibodies (ICA), insulin autoantibodies (IAA), and glutamic acid decarboxylase antibodies (GADA) and insulinoma-like antigen 2 antibodies (IA-2A)]. These autoantibodies were analyzed from serum samples taken at each follow-up visit as previously described (18). ICA levels were determined using an approved, immunofluorescence assay with a detection limit of 2.5 Juvenile Diabetes Foundation Units (JDFU) (19). GADA and IAA levels were quantified using specific radiobinding assays, the threshold of positivity being 5.36 and 3.48 relative units (RU) respectively (20, 21). Similarly, IA-2A levels were measured with a radio-binding assay with a threshold of 0.43 RU (22).

### Analysis of molecular lipids

The samples were randomized and extracted using a modified version of the previously-published Folch procedure (23). 10 µl of serum was mixed with 10 µl 0.9% NaCl and extracted with 120 µl of CHCl_3_: MeOH (2:1, v/v) solvent mixture containing internal standard mixture (c = 2.5 µg/ml).; 1,2-diheptadecanoyl-sn-glycero-3-phosphoethanolamine [PE(17:0/17:0)], N-heptadecanoyl-D-erythro-sphingosylphosphorylcholine [SM(d18:1/17:0)], N-heptadecanoyl-D-erythro-sphingosine [Cer(d18:1/17:0)], 1,2-diheptadecanoyl-sn-glycero-3-phosphocholine [PC(17:0/17:0)], 1-heptadecanoyl-2-hydroxy-sn-glycero-3-phosphocholine [LPC(17:0)] and 1-palmitoyl-d31-2-oleoyl-sn-glycero-3-phosphocholine [PC(16:0/d31/18:1)], cholest-5-en-3ß-yl heptadecanoate [CE(17:0)] and, triheptadecanoylglycerol [TG(17:0/17:0/17:0)].

The samples were vortex mixed and incubated on ice for 30 min after which they were centrifuged at 7800 × g for 5 min. Finally, 60 µL from the lower layer of each sample was collected and mixed with 60 µL of ice-cold CHCl3: MeOH (2:1, *v/v*) in an LC vial.

The Ultra-high performance liquid chromatography-quadrupole time-of-flight mass spectrometry (UHPLC-QTOFMS) analyses were carried out in a similar manner to that described earlier, with some modifications (24, 25). The UHPLC-QTOFMS system was from Agilent Technologies (Santa Clara, CA, USA) combining a 1290 Infinity LC system and 6545 QTOFMS, interfaced with a dual jet stream electrospray (dual ESI) ion source. MassHunter B.06.01 software (Agilent Technologies, Santa Clara, CA, USA) was used for all data acquisition and MZmine 2.53 was used for data processing as described below (26). Identification of lipids was based on in house laboratory LC-MS/MS data on retention time and mass spectra.

Chromatographic separation was performed using an Acquity UPLC BEH C18 column (100 mm × 2.1 mm i.d., 1.7 µm particle size) and a C18 precolumn, both from Waters Corporation (Wexford, Ireland). The mobile phases were water (phase A) and acetonitrile:2-propanol (1:1, *v/v*) (phase B), both containing 1% 1M ammonium acetate and 0.1% (*v/v*) formic acid ammonium acetate as ionization agents. The LC pump was programmed at a flow rate of 0.4 mL min^–1^ and the elution gradient was as follows: from min 0–2, the percentage of phase B was modified from 35% to 80%, from min 2-7, the percentage of phase B was modified from 80% to 100% and then, the final percentage was held for 7 min. A post-time of 7 min was used to regain the initial conditions for the next analysis. Thus, the total analysis time per sample was 21 min (including post processing). The settings of the dual ESI ionization source were as follows: capillary voltage 3.6 kV, nozzle voltage 1500 V, N_2_ pressure in the nebulizer 21 psi, N_2_ flow rate and temperature as sheath gas 11 L min^–1^ and 379°C, respectively. Accurate mass spectra in the MS scan were acquired in the m/z range 100–1700 in positive ion mode.

The peak area obtained for each lipid was normalized with lipid-class-specific internal standards. A (semi)quantitation was performed using lipid-class-specific calibration curves. Pooled samples were used for quality control, in addition to NIST CR-1950 serum and in-house plasma QC serum. The RSD of the concentrations of the identified lipids in QC samples and pooled extracts was on average 6.3% and 16.2%, respectively.

### Analysis of polar metabolites

To a 10 µL aliquot of plasma, 225 µL of ice-cold MeOH (LC-grade, Honeywell) containing the following internal standards (all from Sigma Aldrich): Heptadecanoic acid (5 ppm), DL-valine-d8 (1 ppm), and succinic acid-d4 (1 ppm) was added. The samples were then sonicated in an ice bath for 30 s prior to centrifugation (5500 g, 5 min). 250 µL of the supernatant was transferred to a 2 mL glass auto sampler vial. The pellet was stored at –20°C for protein analysis. The protein content was measured by the Bradford method. The supernatant was dried under a stream of nitrogen at 45°C. Prior to the mass spectrometry measurements, the samples were derivatized using a two-step procedure. Initially the samples were methoximated by incubating the samples with methoxyamine hydrochloride (25 µL, 20mg/mL in pyridine, Sigma Aldrich) at 45°C for 1 h. N-Methyl-N-trimethylsilyltrifluoroacetamide (25 µL, Sigma Aldrich) was then added and the samples were incubated for a further 60 min. A retention index standard containing straight chain, even alkanes (n 10-40, 10 µL, Sigma Aldrich) was added. All this sample prep was performed on a Gerstel robot and timed to the injection into the GC-MS. The derivatized samples were analyzed using gas chromatography (Agilent 7890B) coupled to a time of flight mass spectrometer (Pegasus BT LECO). The metabolites were separated using a 30 m × 0.25 mm (ID) with a film thickness of 0.25 µm HP-5 (Agilent). A guard column (10 m) with an ID of 0.25 mm was used. 1 µL of the sample was injected in split less mode with an inert glass liner (Agilent) held at a temperature of 240°C. The GC was set to constant flow mode (1.2 mL/min) using helium (Aga) as the carrier gas. The GC oven was programed as follows: 50°C (isothermal for 0.2 min), then 7°C/min until 240°C, then 20°C/min until 300°C (isothermal for 5 min). The transfer line was held at 230°C for the whole run. The ion source was set to electron ionization mode and held at 250°C. The MS was scanning from 50 – 650 Da with an extraction rate of 30 kHz and a scan rate of 16 per second.

### Data preprocessing

Lipidomics data processing was performed using open source software MZmine 2.53 (26). The following steps were applied in the processing: 1) Crop filtering with a m/z range of 350 – 1700 m/z and a RT range of 2.0 to 12 min, 2) Mass detection with a noise level of 1200, 3) Chromatogram builder with a minimum time span of 0.08 min, minimum height of 1000 and a m/z tolerance of 0.006 m/z or 10.0 ppm, 4) Chromatogram deconvolution using the local minimum search algorithm with a 70% chromatographic threshold, 0.05 min minimum retention time (RT) range, 5% minimum relative height, 1200 minimum absolute height, a minimum ratio of peak top/edge of 1 and a peak duration range of 0.08 - 5.0, 5) Isotopic peak grouper with a m/z tolerance of 5.0 ppm, RT tolerance of 0.05 min, maximum charge of 2 and with the most intense isotope set as the representative isotope, 6) Join aligner with a m/z tolerance of 0.008 or 10.0 ppm and a weight for of 2, a RT tolerance of 0.1 min and a weight of 1 and with no requirement of charge state or ID and no comparison of isotope pattern, 7) Peak list row filter with a minimum of 12 peaks in a row (= 10% of the samples), 8) Gap filling using the same RT and m/z range gap filler algorithm with an m/z tolerance of 0.006 m/z or 10.0 ppm, 9) Identification of lipids using a custom database search with an m/z tolerance of 0.006 m/z or 10.0 ppm and a RT tolerance of 0.1 min, 10) Normalization using lipid-class-specific internal standards and (semi) quantitation with lipid-class-specific calibration curves, 11) Normalization with total protein amount 12) Data imputation of missing values were done with half of the row’s minimum.

The GC-QMS data was processed in ChromaTOF (v5.51, LECO) using the peak find algorithm. The peak intensities were normalized to heptadecanoic acid. The peaks were manually checked and corrected if needed for correct integration. Metabolites which had a coefficient of variation greater than 30% in the pooled quality control sample or fell below the limit of quantification were excluded from subsequent analysis.

### Statistical methods

The metabolites and lipids data values were log transformed prior to analysis. The difference in the lipidome and metabolome between the studies groups were compared using a multivariate linear model. For longitudinal samples, linear mixed effects model were regressed with fixed effect (~ sex +case + age) and random effect ~ (1 | Subject). For age wise comparisons the lipids/metabolites were regressed with various factors such as sex, and disease conditions (e.g. RP *vs.* SP) using MaAsLin2 package in R (lipids ~ sex +case). To subsequently visualize metabolite level, violin plots from the ggplot2 R package were used.

Pathway analysis of the significant metabolites (p-value < 0.05) was performed in MetaboAnalyst 5.0 (27). The compounds unmatched during compound name matching were excluded from the subsequent pathway analysis. We implemented global test hypergeometric testing for the functional enrichment analysis. The pathway topological analysis was based on the relative betweenness measures of a metabolite in a given metabolic network and for calculating the pathway impact score. Based on the impact values from the pathway topology analysis, the impact value threshold was set to >0.10.

## Results

### Global metabolome and lipidome in rapid vs. slow progressors to type 1 diabetes

We performed untargeted metabolomics and lipidomics analysis in a longitudinal setting among two study groups: 25 rapid progressors who progressed to type 1 diabetes within 18 months after the appearance of the first autoantibody (RP) and 41 slow progressors, who presented with clinical disease after a period of at least 60 month from the initial seroconversion to clinical disease (SP). For each child, samples were analyzed from up to six time points, corresponding to the ages of 3, 6, 12, 18, 24, or ≥36 months (**Figure 1**). The lipidomics dataset (n = 239) included the identified lipids from the following lipid classes: cholesterol esters (CE), ceramides (Cer), diacylglycerols (DG), lysophosphatidylcholines (LPC), phosphatidylcholines (PC), phosphatidylethanolamines (PE), sphingomyelins (SM), and triacylglycerols (TG). Metabolomic dataset (n = 213) included the metabolites from chemical classes, including amino acids, carboxylic acids, phenolic compounds, sugars, and sugar derivatives.

To identify the sources of variation in the lipidomics and metabolomics datasets, multivariable linear model was performed. The concentrations of lipids/metabolites were regressed on factors including age, sex, and case-related phenotypes (*e.g.*, SP *vs*. RP). Among these factors, age showed the strongest effect on the circulating lipidomics and metabolomics profiles. A total of 44 polar metabolites and 106 lipids were related to age (p-value < 0.05, **Supplementary Material SM Tables 1, 2**). A total of 35 out of 175 lipids differed between male and female in the longitudinal setting (p-value < 0.05, **SM Table 3**). However, no sex differences were observed in the metabolomics dataset.

**Figure 2:**
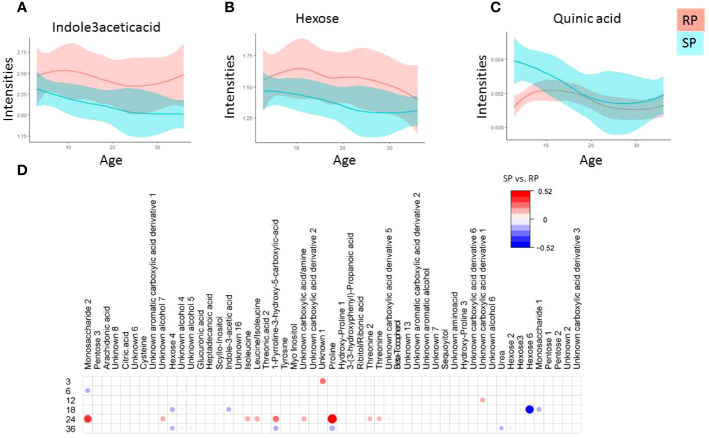
Polar metabolite profiles in serum during the follow-up. **(A-D)** Local polynomial regression fitting (LOESS) curve plot of metabolite concentration over time for the two study groups. Blue, Slow Progressors, SP; red, Rapid Progressors. Solid line, mean value; shaded area, 95% CI. e) Age matched comparison of polar metabolite between SP and RP. The plot shows the most discriminating metabolites between the two study groups compared using multivariable linear model (metabolite ~ sex +case), where case indicate (SP *vs*. RP). Red and blue colors signify linear correlation coefficient up-, down-regulation SP *vs.* RP.

A total of 21 polar metabolites differed between the study groups (SP *vs*. RP; p-value < 0.05, **Figure 2**, **SM Table 4**). The tryptophan-derived microbial catabolite 3-indole acetic acid was downregulated in the SP group (**Figures 2A, B**, p-value <0.05). While most of the sugar derivatives were upregulated in the RP compared to the SP group, quinic acid remained upregulated in the SP group (**Figure 2C**). **Figures 2A-C** shows the local polynomial regression fitting (LOESS) plot of selected metabolite class with time for the case-related phenotypes.

Considering that age is a confounding factor in the metabolomics analysis, we also performed age-matched comparisons between SP and RP (**Figure 2D**). We found that the main metabolic differences between these two groups were after the median age of seroconversion (*i.e.*, around 24-36 months of age). The result showed that at the age of 3, 6, 12, 18, 24 and ≥36 months, a total of 12, 3, 2, 14, 17 and 23, metabolic features were altered, respectively, between the study groups (**Figure 2D**).

Next, the changes in plasma lipid concentration between the study groups in the longitudinal series sample collection were examined. We found that lipid-related differences were less pronounced as compared to polar metabolites. Levels of two lipids, PE (34:0) and TG (18:2/18:1/18:1), were different between SP and RP (p-value < 0.05). With the exception of lipid PE (34:0), there was no consistent lipid related trend between the study groups. The age-wise comparison showed that at the age of 3, 6, 12, 18, 24 or ≥36 months, a total of 1, 1, 1, 13, 3 and 10 lipids were different between the SP and RP groups, respectively (**ESM Figure S1**).

As progression of type 1 diabetes is associated with distinct islet autoantibody trajectories, we compared differences in metabolite changes in crelation to the development of specific islet autoantibodies (IAA *vs*. others). With the exceptions of a carboxylic acid derivative, no other differences (p-value <0.05) between the IAA *vs*. other autoantibodies were observed, which may be due to the small sample size per autoantibody grouping.

### Metabolic pathways discriminating fast *vs*. slow progressors to type 1 diabetes

We then examined the differences between the SP and RP groups at the metabolic pathway level. The selected metabolites/lipids that differed between the case-related phenotypes (SP *vs*. RP) were subjected to metabolic pathway analysis in MetaboAnalyst 5, integrating enrichment-based analysis and pathway topology analysis. Using pathway impact estimates, we found that mainly amino acid -related metabolic processes including phenylalanine, tyrosine and tryptophan biosynthesis, ascorbate and aldarate metabolism, and tyrosine metabolism, pentose and glucuronate interconversions were different between SP and RP (**SM Figure S2**, impact value threshold was set to >0.10).

### Metabolic alterations before and after the first appearance of islet autoantibodies in rapid and slow progressors to type 1 diabetes

**Figure 3:**
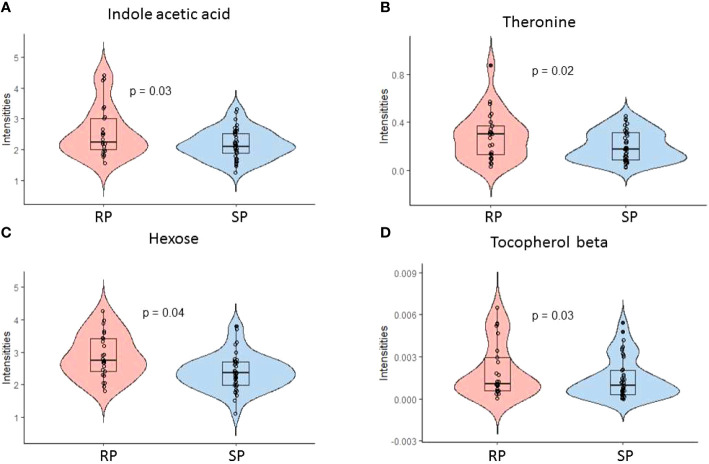
Polar metabolite profiles in serum before and after the first appearance of islet autoantibodies comparing rapid (RP) and slow progressors (SP) to type 1 diabetes. **(A-D)** Violin plot showing the selected discriminating metabolites between the SP and RP. Here, RP and SP is analyzed after the appearance of the first islet autoantibody.

We also studied the metabolic changes between the SP and RP groups (1) prior to the appearance of islet autoantibodies and (2) after the first appearance of islet autoantibodies. We identified differences in polar metabolite profiles after the appearance of the first islet autoantibody when comparing rapid and slow progressors to type 1 diabetes. Altogether, 13 metabolites remained upregulated in rapid progressors as compared to slow progressors (p-value <0.05, **SM Figure S3**). These metabolites can be divided into major chemical classes including sugars, amino acids, and a microbiota-derived metabolite indole-3-acetic acid (*i.e.*, tryptophan catabolite). Among these 13 metabolites, comparative profiles of indole-3-acetic, threonine, beta-tocopherol and hexose are shown in **Figure 3** (p-value <0.05, **ESM Table 5**). We also analyzed metabolite concentration differences between rapid progressors and slow progressors prior to the appearance of islet autoantibodies. Quinic acid and an unknown metabolite were downregulated in rapid progressors when compared to slow progressors before seroconversion (p-value <0.05).

Next, we studied whether lipidomic patterns were related to the development of islet autoantibodies. For that, we analyzed the lipid concentration differences between rapid and slow progressors before and after the emergence of the first islet autoantibody. The results revealed weak differences in lipid levels between the slow and rapid progressors, after or before seroconversion to islet autoantibody positivity. We found two lipid species [PE (O-16:0/22:6) and Cer (d18:1/24:0)] downregulated in slow progressors after the appearance of first islet autoantibodies, when compared to rapid progressors (p-value <0.05). Likewise, there were two lipids [PC (O-38:6) and PE (34:0)] that differed between rapid and slow progressors before the appearance of the first islet autoantibodies (p-value <0.05).

## Discussion

Our study identified specific associations between the metabolic profiles and rate of progression to type 1 diabetes. We observed that specific sugar derivatives and microbial metabolites differed between rapid and slow progressors. While our findings are in line with previous studies showing that metabolic dysregulation precedes islet autoimmunity and type 1 diabetes (11–14), here we showed that after seroconversion to islet autoantibody positivity, slow and rapid progression to clinical diabetes are metabolically different. In agreement with earlier findings (13, 28), we also observed that age was the major confounder in the metabolomics/lipidomics studies.

Recent literature suggests that the gut microbiota is associated with the pathogenesis of type 1 diabetes, however, mechanisms by which gut microbes contribute to the initiation and progression of this disease remain to be identified (29–32). We found that the microbiota-derived metabolite 3-indole-acetic acid was upregulated in children who rapidly progressed to type 1 diabetes following the seroconversion to autoantibody positivity. Previous studies have shown that dysregulated microbial metabolites were associated with the manifestation of type 1 diabetes (33, 34). The microbiota-derived indole metabolites promote intestinal homeostasis and are known for their immunomodulatory signaling activity (34–36). 3-indole-acetic acid is a tryptophan-derived metabolite known to be produced by multiple gut bacterial species, including *Bacteroides, Bifidobacterium, Clostridium* and *Eubacterium* (37). Tryptophan, an essential amino acid, is obtained from dietary protein sources. Increase in tryptophan catabolites (*i.e.*, 3-indole-acetic acid) in circulation suggests a shift from saccharolytic to proteolytic fermentation in the gut among the rapid progressors (38). In line with this, Vatanen et al. showed that microbiota of children who progressed to type 1 diabetes contained less genes that were related to saccharolytic fermentation (29). Thus, the most plausible explanation for the observed elevated 3-indole-acetic acid in the rapid progressors is that the shift in microbial fermentative energy sources dysregulates microbial tryptophan catabolism, which, in turn, exacerbated the pathogenic processes contributing to type 1 diabetes.

Our findings suggest that increasing levels of sugar derivatives in the peripheral circulation (*e.g.*, hexose) contribute to the accelerated progression to type 1 diabetes. The levels of these metabolites were increased in rapid progressors (*vs.* slow) only after the initial appearance of islet autoantibodies. An increased level of sugars in circulation demands higher insulin secretion, leading to beta-cell stress that could contribute to rapid progression to symptomatic type 1 diabetes (39). Recently, in the TRIGR study, Ludvigsson et al. showed that the first appearance of islet cell autoantibody may be related to the increased glucose concentration in blood, corroborating the beta-cell stress hypothesis (40). Intriguingly, Helminen et al. also showed that random plasma glucose predicted the progression to clinical type 1 diabetes in high-risk children (41). We hypothesize that increased insulin demand due to upregulated sugar molecules in the circulation is one of the non-exclusive triggers contributing to the rapid manifestation of type 1 diabetes. Additionally, differences in autoantibody and HLA profiles between RP and SP could affect the individual’s metabolic profile (42). However, we observed minor metabolic dissimilarities linked to the autoantibody profile, particularly between those with IAA presence and those with other autoantibodies. These variances might be due to the small sample size within each autoantibody subgroup.

Growing evidence suggests abnormal lipid metabolism preceding seroconversion to autoantibody positivity and clinical type 1 diabetes (43–46). We also observed changes, albeit subtle, in the levels of phospholipid species in the group of rapid progressors as compared to slow progressors, particularly before seroconversion. However, the earlier studies compared healthy individuals with children *en route* to overt type 1 diabetes, not the rate of progression among the latter subjects.

We acknowledge limitations of this study, one of them being the relatively small sample size in our longitudinal setting. However, to the best of our knowledge, this study presents a novel association of microbiota-derived metabolites, which served as the main discriminators between RP and SP. Next, data on the intestinal microbiome and diet are not available from the study participants. Since microbiota-derived metabolites are crucial in mediating health impacts of the intestinal microbiome, future work could assess the impact of diet–microbe interactions in the progression of type 1 diabetes. Notwithstanding this, our study generates novel hypotheses; however, the causal relationship still remains unknown. With further validation, these findings could advance future research on the disease process leading to clinical type 1 diabetes.

Taken together, our study suggests that a distinct metabolic profile is associated with the rate of progression to type 1 diabetes. The identification of specific metabolites and pathways that differentiate RP from SP may have implications for early intervention strategies to delay or prevent the development of type 1 diabetes.

## Data availability statement

The original contributions presented in the study are included in the article/**Supplementary Material**. Further inquiries can be directed to the corresponding author.

## Ethics statement

The study protocol was approved by the ethics committee of the Hospital District of Southwest Finland. The studies were conducted in accordance with the local legislation and institutional requirements. Written informed consent for participation in this study was provided by the participants’ legal guardians/next of kin.

## Author contributions

Conceptualization: MO, RL, and MKn. Data curation: MKr, PS, and AD. Formal analysis: SL and PS. Funding acquisition: MO, MKn, TH, JT, and RL. Investigation: SL, MO, TH, JT, and MK. Methodology: TH, MKr, and AD. Resources: RV, RL, JT, JI, HH, JL, and MKn. Supervision: TH, MKn, and MO. Writing – first draft: SL. Writing – critical review & editing: all authors. All authors contributed to the article and approved the submitted version.

## References

[1] Katsarou A Gudbjörnsdottir S Rawshani A Dabelea D Bonifacio E Anderson BJ. Type 1 diabetes mellitus. Nat Rev Dis Primers (2017) 3:17016. doi: 10.1038/nrdp.2017.16 28358037

[2] Knip M Veijola R Virtanen SM Hyöty H Vaarala O Akerblom HK. Environmental triggers and determinants of type 1 diabetes. Diabetes (2005) 54 Suppl 2:S125–36. doi: 10.2337/diabetes.54.suppl_2.S125 16306330

[3] Rewers M Hyöty H Lernmark Å Hagopian W She JX Schatz D. The environmental determinants of diabetes in the young (TEDDY) study: 2018 update. Curr Diabetes Rep (2018) 18:136. doi: 10.1007/s11892-018-1113-2 PMC6415767 30353256

[4] Predieri B Bruzzi P Bigi E Ciancia S Madeo SF Lucaccioni L. Endocrine disrupting chemicals and type 1 diabetes. Int J Mol Sci 21 (2020) 21(8):293. doi: 10.3390/ijms21082937 PMC7215452 32331412

[5] Ziegler AG Rewers M Simell O Simell T Lempainen J Steck A. Seroconversion to multiple islet autoantibodies and risk of progression to diabetes in children. JAMA (2013) 309:2473–9. doi: 10.1001/jama.2013.6285 PMC4878912 23780460

[6] Jia X Gu Y High H Yu L. Islet autoantibodies in disease prediction and pathogenesis. Diabetol Int (2020) 11:6–10. doi: 10.1007/s13340-019-00414-9 31949998 PMC6942067

[7] Anand V Li Y Liu B Ghalwash M Koski E Ng K. Islet autoimmunity and HLA markers of presymptomatic and clinical type 1 diabetes: joint analyses of prospective cohort studies in Finland, Germany, Sweden, and the U.S. Diabetes Care (2021) 44:2269–76. doi: 10.2337/dc20-1836 PMC8929180 34162665

[8] Achenbach P Hummel M Thümer L Boerschmann H Höfelmann D Ziegler AG. Characteristics of rapid vs slow progression to type 1 diabetes in multiple islet autoantibody-positive children. Diabetologia (2013) 56:1615–22. doi: 10.1007/s00125-013-2896-y 23539116

[9] Steck AK Johnson K Barriga KJ Miao D Yu L Hutton JC. Age of islet autoantibody appearance and mean levels of insulin, but not GAD or IA-2 autoantibodies, predict age of diagnosis of type 1 diabetes: diabetes autoimmunity study in the young. Diabetes Care (2011) 34:1397–9. doi: 10.2337/dc10-2088 PMC3114355 21562325

[10] Steck AK Vehik K Bonifacio E Lernmark A Ziegler AG Hagopian WA. Predictors of progression from the appearance of islet autoantibodies to early childhood diabetes: The environmental determinants of diabetes in the young (TEDDY). Diabetes Care (2015) 38:808–13. doi: 10.2337/dc14-2426 PMC4407751 25665818

[11] Lamichhane S Kemppainen E Trošt K Siljander H Hyöty H Ilonen J. Circulating metabolites in progression to islet autoimmunity and type 1 diabetes. Diabetologia (2019) 62:2287–97. doi: 10.1007/s00125-019-04980-0 PMC6861356 31444528

[12] Oresic M Simell S Sysi-Aho M Nanto-Salonen K Seppanen-Laakso T Parikka V. Dysregulation of lipid and amino acid metabolism precedes islet autoimmunity in children who later progress to type 1 diabetes. J Exp Med (2008) 205:2975–84. doi: 10.1084/jem.20081800 PMC2605239 19075291

[13] Lamichhane S Ahonen L Dyrlund TS Kemppainen E Siljander H Hyöty H. Dynamics of plasma lipidome in progression to islet autoimmunity andtype 1 diabetes - type 1 diabetes prediction and prevention Study (DIPP). Sci Rep (2018) 8:10635. doi: 10.1038/s41598-018-28907-8 30006587 PMC6045612

[14] Sen P Dickens AM López-Bascón MA Lindeman T Kemppainen E Lamichhane S. Metabolic alterations in immune cells associate with progression to type 1 diabetes. Diabetologia (2020) 63:1017–31. doi: 10.1007/s00125-020-05107-6 PMC7145788 32043185

[15] Kupila A Muona P Simell T Arvilommi P Savolainen H Hamalainen AM. Juvenile Diabetes Research Foundation Centre for the Prevention of Type, Feasibility of genetic and immunological prediction of type I diabetes in a population-based birth cohort. Diabetologia (2001) 44:290–7. doi: 10.1007/s001250051616 11317658

[16] Ilonen J Reijonen H Herva E Sjoroos M Iitia A Lovgren T. Rapid HLA-DQB1 genotyping for four alleles in the assessment of risk for IDDM in the Finnish population. The Childhood Diabetes in Finland (DiMe) Study Group. Diabetes Care (1996) 19:795–800. doi: 10.2337/diacare.19.8.795 8842593

[17] Ilonen J Kiviniemi M Lempainen J Simell O Toppari J Veijola R. Genetic susceptibility to type 1 diabetes in childhood - estimation of HLA class II associated disease risk and class II effect in various phases of islet autoimmunity. Pediatr Diabetes (2016) 17 Suppl 22:8–16. doi: 10.1111/pedi.12327 27411431

[18] Siljander HT Simell S Hekkala A Lähde J Simell T Vähäsalo P. Predictive characteristics of diabetes-associated autoantibodies among children with HLA-conferred disease susceptibility in the general population. Diabetes (2009) 58:2835–42. doi: 10.2337/db08-1305 PMC2780879 19755526

[19] Bottazzo GF Florin-Christensen A Doniach D. Islet-cell antibodies in diabetes mellitus with autoimmune polyendocrine deficiencies. Lancet (1974) 2:1279–83. doi: 10.1016/S0140-6736(74)90140-8 4139522

[20] Savola K Sabbah E Kulmala P Vahasalo P Ilonen J Knip M. Autoantibodies associated with Type I diabetes mellitus persist after diagnosis in children. Diabetologia (1998) 41:1293–7. doi: 10.1007/s001250051067 9833935

[21] Ronkainen MS Hamalainen AM Koskela P Akerblom HK Knip M. Pregnancy induces nonimmunoglobulin insulin-binding activity in both maternal and cord blood serum. Clin Exp Immunol (2001) 124:190–6. doi: 10.1046/j.1365-2249.2001.01506.x PMC1906063 11422194

[22] Savola K Bonifacio E Sabbah E Kulmala P Vahasalo P Karjalainen J. IA-2 antibodies–a sensitive marker of IDDM with clinical onset in childhood and adolescence. Childhood Diabetes in Finland Study Group. Diabetologia (1998) 41:424–9. doi: 10.1007/s001250050925 9562346

[23] Folch J Lees M Sloane Stanley G. A simple method for the isolation and purification of total lipids from animal tissues. J Biol Chem (1957) 226:497–509. doi: 10.1016/S0021-9258(18)64849-5 13428781

[24] Nygren H Seppanen-Laakso T Castillo S Hyotylainen T Oresic M. Liquid chromatography-mass spectrometry (LC-MS)-based lipidomics for studies of body fluids and tissues. Methods Mol Biol (2011) 708:247–57. doi: 10.1007/978-1-61737-985-7_15 21207295

[25] Pedersen HK Forslund SK Gudmundsdottir V Petersen AO Hildebrand F Hyotylainen T. A computational framework to integrate high-throughput '-omics' datasets for the identification of potential mechanistic links. Nat Protoc (2018) 13:2781–800. doi: 10.1038/s41596-018-0064-z 30382244

[26] Pluskal T Castillo S Villar-Briones A Oresic M. MZmine 2: modular framework for processing, visualizing, and analyzing mass spectrometry-based molecular profile data. BMC Bioinform (2010) 11:395. doi: 10.1186/1471-2105-11-395 PMC2918584 20650010

[27] Pang Z Chong J Zhou G de Lima Morais DA Chang L Barrette M. MetaboAnalyst 5.0: narrowing the gap between raw spectra and functional insights. Nucleic Acids Res (2021) 49:W388–w396. doi: 10.1093/nar/gkab382 34019663 PMC8265181

[28] Rist MJ Roth A Frommherz L Weinert CH Krüger R Merz B. Metabolite patterns predicting sex and age in participants of the Karlsruhe Metabolomics and Nutrition (KarMeN) study. PloS One (2017) 12:e0183228. doi: 10.1371/journal.pone.0183228 28813537 PMC5558977

[29] Vatanen T Franzosa EA Schwager R Tripathi S Arthur TD Vehik K. The human gut microbiome in early-onset type 1 diabetes from the TEDDY study. Nature (2018) 562:589–94. doi: 10.1038/s41586-018-0620-2 PMC6296767 30356183

[30] Vatanen T Kostic AD d'Hennezel E Siljander H Franzosa EA Yassour M. Variation in microbiome LPS immunogenicity contributes to autoimmunity in humans. Cell (2016) 165:842–53. doi: 10.1016/j.cell.2016.04.007 PMC4950857 27133167

[31] Knip M Siljander H. The role of the intestinal microbiota in type 1 diabetes mellitus. Nat Rev Endocrinol (2016) 12:154–67. doi: 10.1038/nrendo.2015.218 26729037

[32] Zheng P Li Z Zhou Z. Gut microbiome in type 1 diabetes: A comprehensive review. Diabetes Metab Res Rev (2018) 34:e3043. doi: 10.1002/dmrr.3043 29929213 PMC6220847

[33] Galderisi A Pirillo P Moret V Stocchero M Gucciardi A Perilongo G. Metabolomics reveals new metabolic perturbations in children with type 1 diabetes. Pediatr Diabetes (2018) 19:59–67. doi: 10.1111/pedi.12524 28401628

[34] Balderas C Rupérez FJ Ibañez E Señorans J Guerrero-Fernández J Casado IG. Plasma and urine metabolic fingerprinting of type 1 diabetic children. Electrophoresis (2013) 34:2882–90. doi: 10.1002/elps.201300062 23857511

[35] Hubbard TD Murray IA Bisson WH Lahoti TS Gowda K Amin SG. Adaptation of the human aryl hydrocarbon receptor to sense microbiota-derived indoles. Sci Rep (2015) 5:12689. doi: 10.1038/srep12689 26235394 PMC4522678

[36] Alexeev EE Lanis JM Kao DJ Campbell EL Kelly CJ Battista KD. Microbiota-derived indole metabolites promote human and murine intestinal homeostasis through regulation of interleukin-10 receptor. Am J Pathol (2018) 188:1183–94. doi: 10.1016/j.ajpath.2018.01.011 PMC5906738 29454749

[37] Roager HM Licht TR. Microbial tryptophan catabolites in health and disease. Nat Commun (2018) 9:3294. doi: 10.1038/s41467-018-05470-4 30120222 PMC6098093

[38] Windey K De Preter V Verbeke K. Relevance of protein fermentation to gut health. Mol Nutr Food Res (2012) 56:184–96. doi: 10.1002/mnfr.201100542 22121108

[39] Ludvigsson J. Why diabetes incidence increases–a unifying theory. Ann N Y Acad Sci (2006) 1079:374–82. doi: 10.1196/annals.1375.058 17130582

[40] Ludvigsson J Cuthbertson D Becker DJ Kordonouri O Aschemeier B Pacaud D. Increasing plasma glucose before the development of type 1 diabetes-the TRIGR study. Pediatr Diabetes (2021) 22:974–81. doi: 10.1111/pedi.13251 PMC8530903 34369627

[41] Helminen O Aspholm S Pokka T Ilonen J Simell O Veijola R. OGTT and random plasma glucose in the prediction of type 1 diabetes and time to diagnosis. Diabetologia (2015) 58:1787–96. doi: 10.1007/s00125-015-3621-9 25985749

[42] Pöllänen PM Lempainen J Laine AP Toppari J Veijola R Vähäsalo P. Characterisation of rapid progressors to type 1 diabetes among children with HLA-conferred disease susceptibility. Diabetologia (2017) 60:1284–93. doi: 10.1007/s00125-017-4258-7 28364254

[43] Balzano-Nogueira L Ramirez R Zamkovaya T Dailey J Ardissone AN Chamala S. Integrative analyses of TEDDY omics data reveal lipid metabolism abnorMalities, increased intracellular ROS and heightened inflammation prior to autoimmunity for type 1 diabetes. Genome Biol (2021) 22:39. doi: 10.1186/s13059-021-02262-w 33478573 PMC7818777

[44] Afshinnia F RajendIran TM He C Byun J Montemayor D Darshi M. Circulating free fatty acid and phospholipid signature predicts early rapid kidney function decline in patients with type 1 diabetes. Diabetes Care (2021) 44:2098–106. doi: 10.2337/dc21-0737 PMC8740931 34244329

[45] Holm LJ Krogvold L Hasselby JP Kaur S Claessens LA Russell MA. Abnormal islet sphingolipid metabolism in type 1 diabetes. Diabetologia (2018) 61:1650–61. doi: 10.1007/s00125-018-4614-2 PMC6445476 29671030

[46] Oresic M. Metabolomics in the studies of islet autoimmunity and type 1 diabetes. Rev Diabetes Stud (2012) 9:236–47. doi: 10.1900/RDS.2012.9.236 PMC3740693 23804263

