# Supplementary material for: Circulating metabolic signatures of rapid and slow progression to type 1 diabetes in islet autoantibody-positive children
Source: Front Endocrinol (Lausanne). 2023 Sep 6;14:1211015. doi: 10.3389/fendo.2023.1211015 (PMC10516565; doi:10.3389/fendo.2023.1211015)
Supplement: Supplementary file 2 [file DataSheet_1.docx]

**Fig. S1 Plasma lipid concentration differences between the study groups (SP vs RP) in the longitudinal series sample set.**


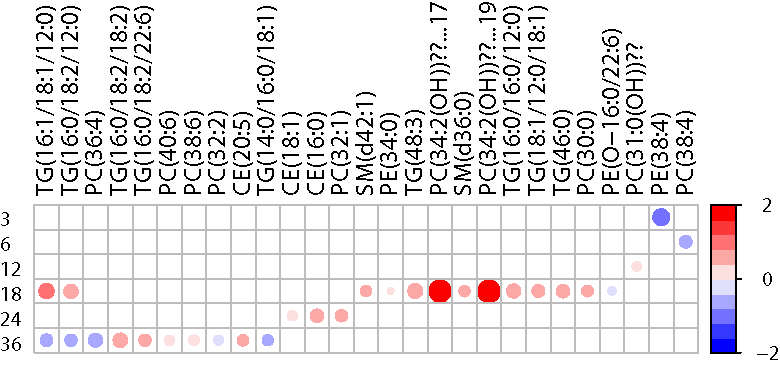


**Fig. S2 Metabolic pathways discriminating slow *vs*. fast progressors to type 1 diabetes**

**
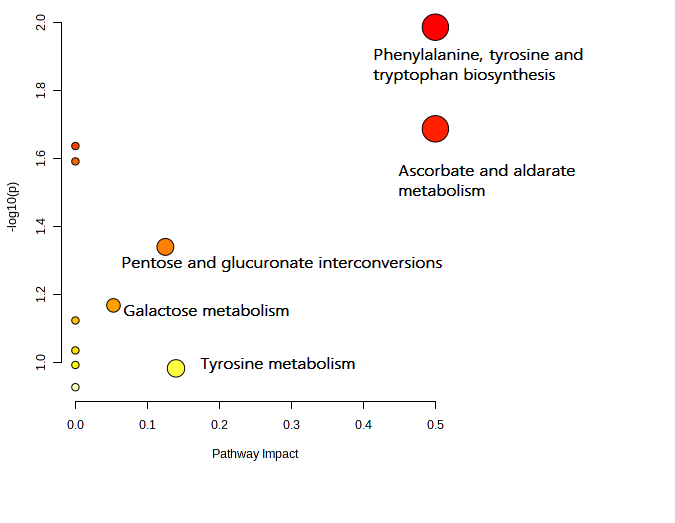
**

**Fig. S3 Metabolic alterations before and after the first appearance of islet autoantibodies in rapid and slow progressors to type 1 diabetes**

**
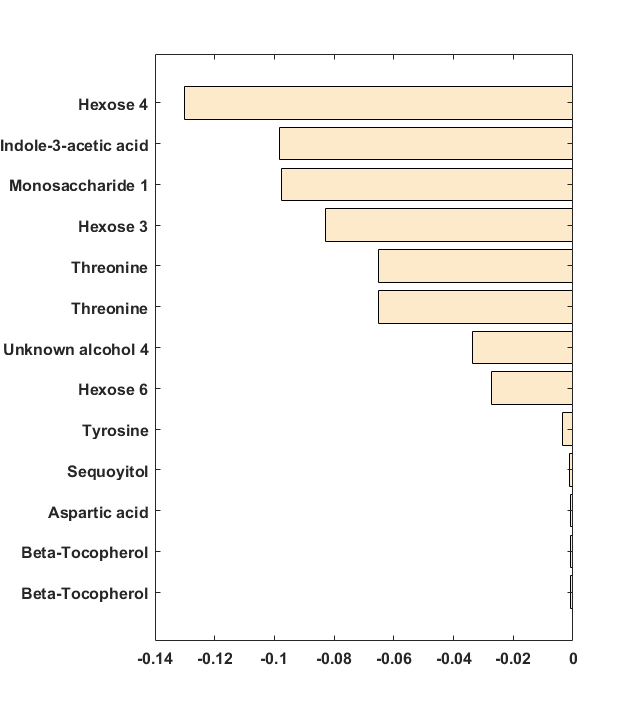
**
